# The changes and prognostic value of liver function in young adults with severe burn: A retrospective observational study

**DOI:** 10.1097/MD.0000000000013721

**Published:** 2018-12-21

**Authors:** Yan Gong, Xianming Long, Hua Xu, Xinjing Yang, Qiang Guo

**Affiliations:** a Department of Rehabilitation medicine, The Affiliated Suzhou Hospital of Nanjing Medical University; b Department of Emergency and Critical Care Medicine, The First Affiliated Hospital of Soochow University; c Department of Rheumatology and Immunology, The First Affiliated Hospital of Soochow University, Suzhou, Jiangsu, China.

**Keywords:** burn, critical care, Liver dysfunction

## Abstract

To analyze the changes in liver functions and the relationship between alterations in liver function and mortality risk in young adults with third-degree burn wounds on over 90% of the total body surface area (TBSA).

A total of 23 fatally burned factory workers in an inflammable dust explosion and fire were enrolled from 2 intensive care units. Clinical data, particularly the laboratory tests for liver function, were retrospectively analyzed and compared between the survivor and non-survivor groups.

Compared to survivors, non-survivors had significantly higher total bilirubin (TBIL), glutamate-pyruvate transaminase (GPT), glutamic-oxaloacetic transaminase, alkaline phosphatase, prothrombin time, and activated partial thromboplastin time (APTT) at the terminal point of this study (*P* <.05). In addition, the peak values of TBIL, GPT, and longer APTT were higher in non-survivors than in survivors during hospital course, and the peak values of TBIL was one of major prognostic factors for mortality risk. Furthermore, at the first 2 weeks, the cumulative survival rates were significantly lower in patients with liver dysfunction than those without liver dysfunction (*P* <.01).

Our findings show that the great changes in liver function occurred in first 2 weeks after severe burns. Liver dysfunction may have an effect on clinical outcomes of post-burn. Measures to protect liver function and prevent from deterioration could be beneficial in improvement survival rate, especially during the first 2 weeks.

## 1 Introduction

Extensive burn represents a devastating traumatic injury and leads to high mortality rates in affected patients. The last 2 decades have witnessed a number of advances in burn research and therapeutic strategies for the treatment of severely burned patients, including improved fluid resuscitation, infection control, would healing, new grafting materials, the management of inhalation injuries, and life support techniques for the clinical care for burns, which have significantly improved post-burn clinical outcomes.^[1,2]^ However, both mortality and morbidity rates have been reported to remain high among patients with severe burns. Therefore, there is an urgent need to identify mortality-related causative factors underlying the low survival rates in patients with severe burns.

Liver damage is one of the most common forms of post-burn complications. In fact, the liver has been reported to play a pivotal role in pathophysiological processes in response to thermal injury, which includes metabolic functions, inflammatory processes, and immune regulation.^[3–6]^

However, the exact functional role of the liver in post-burn response and the association of this important organ with post-burn survival are not largely understood. It has been proposed that liver homeostasis could be essential for survival and clinical outcomes following severe burns. However, the study of the association between liver damage and the survival of adult patients with extensive burns has been hampered due to limited data. According to the World Health Organization (WHO), it is estimated that 322,000 deaths per year in the globe are correlated with thermal injury.^[7]^ However, a group of young severely burned patients with third-degree burns and burn wounds in >90% of the total body surface area (TBSA) due to the same flammable dust explosion and fire represent a rare case for burn study.

In this retrospective study, a total of 23 fatally burned factory workers in an inflammable dust explosion and fire in Kunshan, China, who were admitted to two intensive care units of the Affiliated Suzhou Hospital of Nanjing Medical University and the First Affiliated Hospital of Soochow University, were enrolled in the present retrospective study, Clinical data, particularly the laboratory tests for the liver function of survivors *vs* non-survivors, were collected and analyzed. We intended to analyze the changes in liver functions and the relationship between alterations in liver function and mortality risk in young adults with third-degree burn and >90% of TBSA.

## 2 Study subjects and methods

### 2.1 Subjects and designs

In the present study, a total of 23 young factory workers, who were severely injured in an accident of flammable dust explosion and fire at a factory in Kunshan City, Jiangsu Province in China, were admitted to 2 critical care units of Suzhou Municipal Hospital and the First Affiliated Hospital of Soochow University and received the same treatment at 2 centers. All burned patients received standard of care, including surfactant therapy and immunoglobulin treatment in hospital.

Upon admission to the intensive care units, all patients received the same standard of care for acute-burns and supportive treatment with the renal replacement and mechanical ventilation. In brief, tracheotomy and adequacy of fluid resuscitation therapy were initiated at the same time and the patients were closely monitored with a large volume of blood plasma and other blood products provided across the country. After admission, total burn wound debridement was immediately performed. For the closure of extensive deep burn wounds, intermingled transplantation using techniques including autograft, allograft, and micrograft were performed according to the clinical condition of each patient. This procedure was repeatedly undertaken until all areas of open wound were covered with autologous skin.

Airway management consisted of immediate tracheostomy, rotating bed, postural drainage, intra-bronchial rinse, and the inhalation of exogenous surfactants. In order to assess the degree of inhalation injury (INFI), fiber bronchoscopy was performed for all patients. The degree of INFI was defined by the grading system of INHI (0, 1, 2, 3, and 4), which was derived from findings at the initial bronchoscopy and based on Abbreviated Injury Score (AIS) criteria as described previously described.^[8]^ Mechanical ventilation was initially performed using the modes of synchronized intermittent mandatory or pressure control, followed by pressure support ventilation with positive end-expiratory pressure or inspired oxygen as the patient's requirements lessened. Continuous renal replacement therapy (CRRT) was applied in the patients who developed acute kidney injury (AKI), rhabdomyolysis, electrolyte disorder or severe acidosis. At the same time, early enteral nutritional support via a naso-duodenal or naso-gastric tube was initiated. All patients were given the same nutritional supplement.

These patients were followed up for 1-year post-hospitalization, during which 11 patients were survived, and 12 patients died and the deaths with death occurred during the first 3 months (90 days). Therefore, the first 90 days following the severe burn were selected as the data period, with different time points of 30, 60 and 90 days in the present retrospective study. The highest mortality rate was observed during the first 2 weeks after injured.

### 2.2 Inclusion criteria

All patients were diagnosed with third-degree burns and a total burn wound area of >90% TBSA (≥90% TBSA), which were defined as extensive burns in the present study.

### 2.3 Exclusion criteria

They were generally young adults and had significant medical history (e.g., viral hepatitis, liver fibrosis, lung disease, or kidney disease). Moreover, all the patients had concurrent viral hepatitis during the treatment period were excluded from the present study. In addition, Patients who had been treated for any liver damage during the treatment were also excluded.

### 2.4 Patient consent and standard protocol approval

All patients provided a written informed consent to participate in the present study and signed additional consent forms to agree with the use of their information and data for scientific purposes. The study was reviewed and approved by the Institutional Review Board (IRB) of the First Affiliated Hospital of Soochow University and the Affiliated Suzhou Hospital of Nanjing Medical University.

## 3 Methods

### 3.1 Demographics and collection of clinical data

In order to determine whether the changes in the liver function or hepatic dysfunction could contribute to the increase in mortality, the study subjects were divided into 2 groups: survivor group and non-survivor group. Baseline demographic and clinical information, including age, gender, burn size, depth of burn, acute physiology, and chronic health evaluation (APACHE II) score and sequential organ failure assessment (SOFA) score were collected as previously reported.^[9]^ Concomitant injuries such as INFI, sepsis, morbidity, and mortality were also recorded during the 90 days after admission to hospital. Sepsis was defined and diagnosed according to previously published criteria.^[10]^ The incidence of septic shock, acute respiratory distress syndrome (ARDS), AKI and acute gastrointestinal injury (AGI), thrombocytopenia (platelet count <20,000 per mm^3^) and liver dysfunction^[11]^ were also recorded in the different study groups.

### 3.2 Evaluations for liver function

Laboratory tests for liver function and injury, which included total bilirubin (TBIL) of both conjugated and unconjugated bilirubin, glutamate pyruvate transaminase, glutamic-oxal(o)acetic transaminase, alkaline phosphatase (ALP), prothrombin time (PT), activated partial thromboplastin time (APPT), were conducted at different time points in the patients, and data were collected and analyzed for Index of Liver function. liver disfunction was defined as the value of ALT (AST) increased by 5 times of upper limit of normal (ULN), ALP increased by 5 times of ULN and TBIL increased by 1.5 times of ULN.^[11]^

### 3.3 Statistical analysis

IBM SPSS statistical software version 17.0 (Chicago, IL) was used for statistical analysis in the present study. All data were expressed as mean ± standard deviation (SD), or median and interquartile range. Categorical data were presented as frequency (percentage). The comparisons of variables between the survivor and non- survivor groups were performed using Student's *t*-test or Mann–Whitney *U* test for continuous data, and Chi-square test or Fisher Exact test for categorical data. The survival curves were generated using the Kaplan–Meier survival rate estimates between the liver failure and non-liver failure groups. Multivariate Cox proportional hazard regression was performed to assess the independent contributors to mortality of each potential risk factor. *P* <.05 was considered statistical significantly.

## 4 Results

### 4.1 Demographic and clinical characteristics of patients

According to above inclusion criteria and exclusion criteria, a total of 23 patients with third-degree burns induced by inflammable dust explosion and fire, were eligible to participate into the present study from 2 intensive care units. The mean age of these patients was 38.13 ± 7.55 years old. Among the 23 studied patients, 16 (69.56%) patients were male, and 7 (30.44%) patients were female. The mean burn TBSA was 95.5 ± 2.5%, the mean third-degree burn areas was >90%, and APACHE II and SOFA scores respectively were 18.8 ± 3.2 and 12.6 ± 3.1.

Upon admission to the intensive care units, these patients were given the same standard of treatment for acute burns and supportive clinical care. The mean daily volume of blood plasma during the hospitalization was 1308.4 ± 265.4 mL. In the present study, a mean fluid volume of 1315.60 mL was administered within the first 24 hours. The first half of the fluid volume was given during the first 8 hours, while the remaining amount was given within the next 16 hours. A large volume of blood plasma was provided for each patient, especially during the acute phase of fetal burns. In the first week after hospitalization, the mean daily volume of blood plasma reached up to 2400 mL per patient.

A total of 12 patients died within 90 days from time of admission to the hospital, and another 11 patents were survived in severe burns. The baseline demographic and clinical characteristics were compared between non-Survivors group and Survivors group, which were summarized in Table 1. The 2 groups had similar demographic and baseline clinical characteristics, and great differences not were observed in 2 groups with respect to age, gender, burn TBSA, Acute Physiology and Chronic Health Evaluation (APCHEH) II score. Furthermore, there were no significant differences in complications, including ARDS, AKI, AGI, sepsis, and INFI. The basic therapeutic measures were also not significant differences, For example, the coverage of the autodermic graft, the daily volume of blood plasma, ventilation dependency, and the duration of CRRT.

**Table 1:**
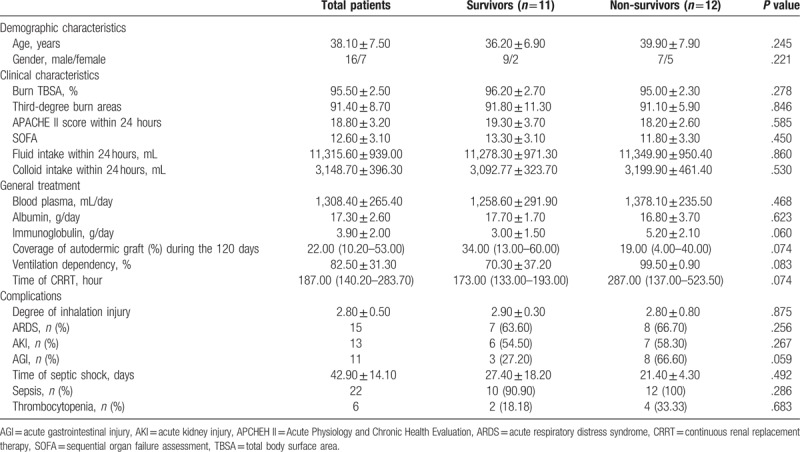
Demographic and clinical characteristics between survivors and non-survivors.

### 4.2 The changes in liver function at different time points during hospitalization

**Figure 1:**
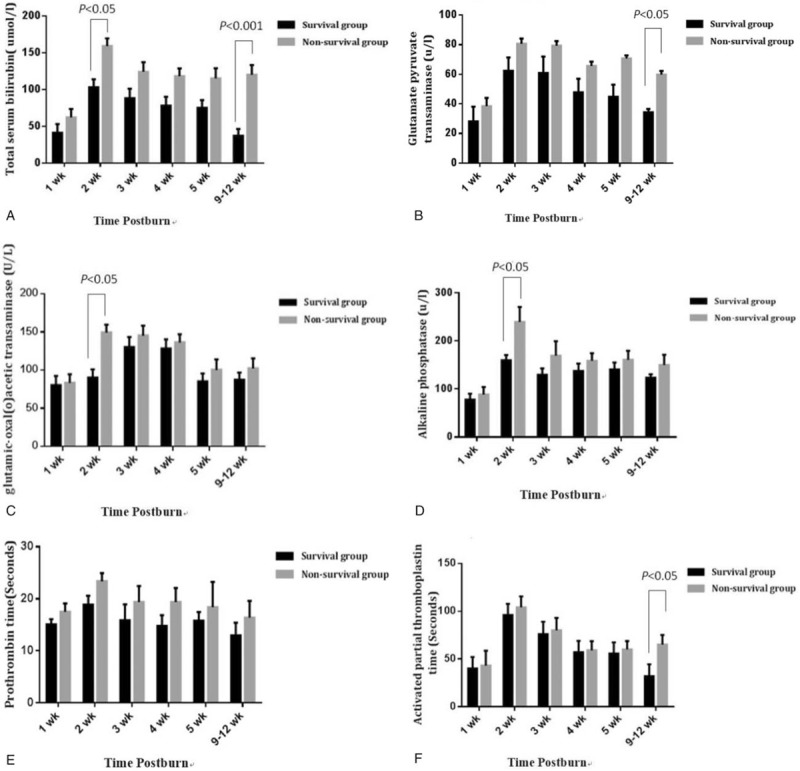
The comparative analysis of liver function in the survivor and non-survivor groups at different time points following burns. (A) Total serum bilirubin; (B) Glutamate pyruvate transaminase; (C) Glutamic-oxaloacetic transaminase; (D) Alkaline phosphatase; (E) Prothrombin time; (F) Activated partial thromboplastin time.

To explore the association of changes in liver function with mortality risk, we analyzed the changes in liver function at different time points of hospitalization between survivors and non-survivors. The main results were summarized in Table 2. Differences in background liver function at the time of admission between patients in the survivor and non-survivor groups were not statistically significant. However, the peak value of TBIL was significantly greater in the non-survivor group compared to the survivor group. In addition, glutamic-oxaloacetic transaminase (GOT) levels, PT, and APPT at the endpoint during the hospital course were higher in the non-survivor group (*P* <.05) (Fig. 1). Furthermore, it was also found that changes in total serum TBIL, glutamate pyruvate transaminase, GOT, ALP, PT, and APPT had the same trend in the survivor group, and the values of all liver function markers in the non-survivor group at each time point were higher than those in the survivor group. Moreover, the peak values of these markers, except for PT, occurred at week 2 after hospitalization. Non-survivors had significantly higher peak values of TBIL and glutamate pyruvate transaminase, and longer APPT at the terminal point, compared to that at the same time point in survivors. In particular, these markers were maintained at high levels for 11 weeks (from week 2–12) in the non-survival group. Furthermore, the most significant differences in liver function between survivors and non-survivors were detected at the first 2 weeks.

**Table 2:**
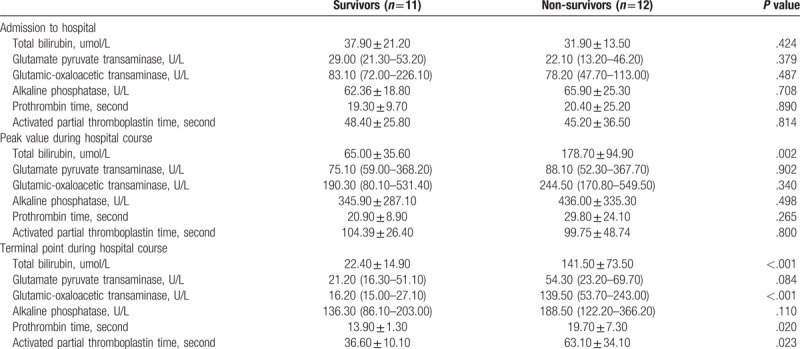
Changes in liver function at different time points during hospitalization between survivors and non-survivors.

In order to exclude the possibility that blood plasma could influence liver function in the present study, the associations of blood plasma were further analyzed with the indices of liver function through a correlation analysis. No significant correlation was observed between blood plasma and the indices of liver function (Table 3).

**Table 3:**
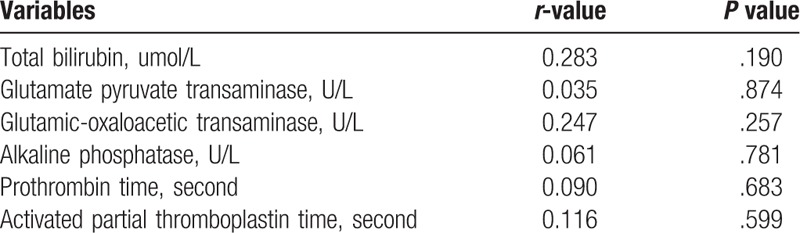
Correlation analysis of the amount of plasma and changes in liver function.

### 4.3 The association of TBIL with mortality

**Figure 2:**
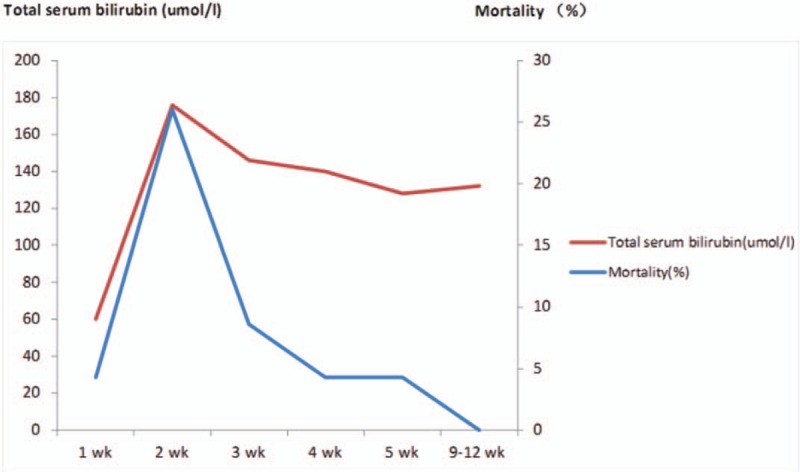
The correlation of mortality rates with total serum bilirubin levels in the non-survivor group. Serial mean serum levels of total bilirubin and mortality rates at different observation periods (1, 2, 3, 4, 5, and 9–12 week) were measured and illustrated.

**Figure 3:**
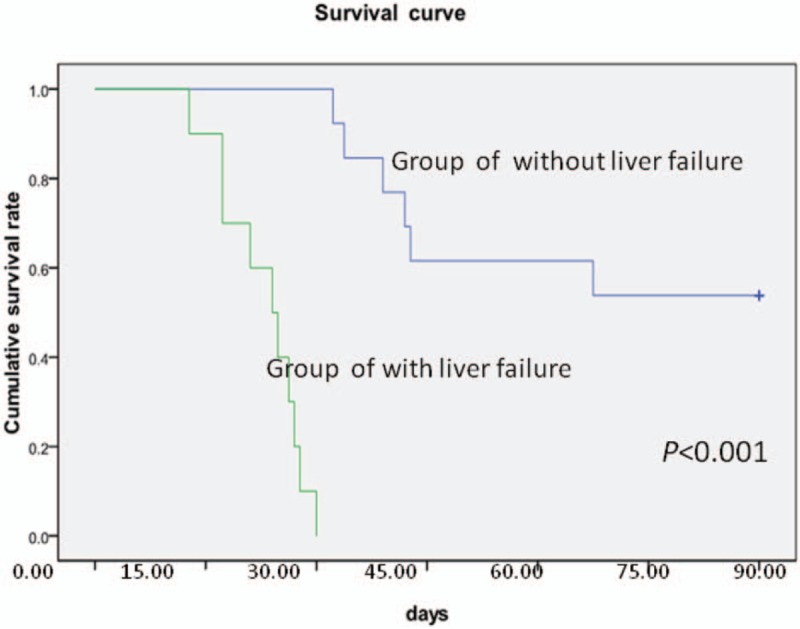
The analysis of the cumulative survival rates of patients with or without liver dysfunction throughout the 90-day observation period.

To further explore the association between mortality risk and the TBIL of non-survivors, the data were subjected to correlation analysis. As shown in Figure 2, the change in mortality rates was roughly parallel to the alteration in TBIL among non-survivors. Kaplan-Meier survival analysis was also performed to compare the cumulative survival rates of patients with or without liver dysfunction, and it was found that the survival rate of patients without liver dysfunction was significantly greater than patients with liver dysfunction during hospitalization (*P* <.001, Fig. 3).

### 4.4 Multivariate analysis of independent factors for predicting mortality risk

In order to identify the independent factors for predicting mortality risk during follow-up, a multivariate analysis determined by Cox proportional hazards stepwise regression was performed. These results were shown in Table 4. APACHE II score within 24 hours and the peak values of TBIL were the major prognostic factors for mortality risk of severe burn.

**Table 4:**
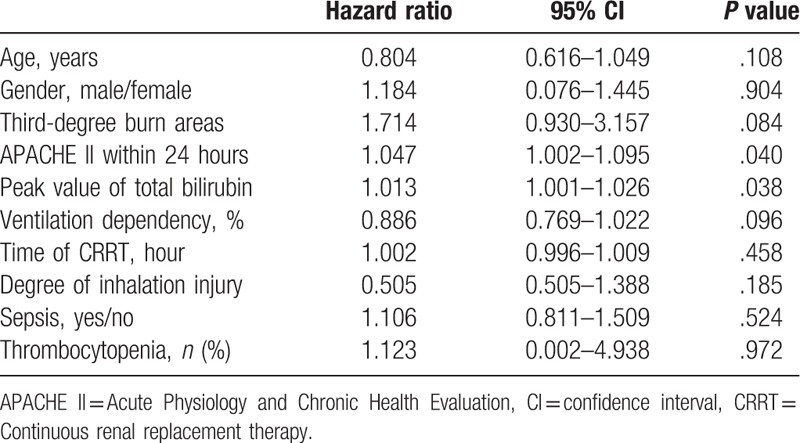
Multivariate analysis of independent factors for predicting mortality risk.

## 5 Discussion

Bum was a kind of complex disease caused by heat, chemicals, current, or radiation. It was not only a common trauma in daily life and work but also was one of the major war wounds. The severity of burn damage main influenced by bum area and depth except for individual factors.

Despite the considerable advances in critical care and the management of burns, mortality rates remain high in severely burned patients. It has been well-recognized that burn size is one of the most important factors that affect the survival rates of adult patients with extensive burns, and both morbidity and mortality increase in response to the increase in burn area. A recent study has reported that the mortality rate of patients with burn wounds >70% TBSA reached as high as 69%.^[12]^ In the present study, it is noteworthy that all patients had burn areas >90% TBSA, while the mortality rate (12/23, 52%) was lower than that previously reported.^[12,13]^ This is likely attributed to the short hospital admission time and treatment-related factors, including the availability of large blood products, supportive techniques, and the surgical procedure.^[14,15]^ First, in the present study, all burn patients were immediately sent to intensive care units as quickly as possible, and most of these patients were admitted to the intensive units within 8 hours. Second, the investigator's strong technical support and advances in the fields of resuscitation, hypermetabolism, wound healing, grafting techniques and materials, pulmonary and renal support, and infection and monitoring methods in the intensive care unit of our hospital. Third, a large volume of blood products was provided for the patients, and at the first weeks following hospital admission, the daily volume of blood plasma used for each patient reached as high as 2400 mL. Lastly, surgical procedures of the early excision of devitalized tissues and prompt wound closure were performed for these burned patients, since early excision within 72 hours has been found to reduce patient mortality,^[14,16]^ and severely burn patients would benefit from early excision and dermatoplasty.

In addition to the percentage of TBSA, other factors such as INFI, liver function, serum creatinine level, inotropic support, platelet count, sepsis, and ventilator dependency have also been identified to be significantly associated with increased in mortality risk.^[6,17]^ These results, which were obtained from patients with similar demographic and clinical characteristics in an accident of dust explosion and fire, confirm the importance of liver function in the survival of patients with severe burns. In the present study, it was found that only the peak values of TBIL and the APACHE II score were independent predictors for mortality risk. Moreover, liver dysfunction may independently contribute to increased mortality in young adult patients with third-degree burns and wounds >90% TBSA. According to previous study,^[18]^ these results may be associated with invertible hepatocyte necrosis in burn injuries.

In the present study, it was observed that TBIL, glutamate pyruvate transaminase, GOT, ALP, and APPT drastically increased from week 2 to week 12, which matched the changes in mortality and TBIL in non-survivors. These findings suggest that liver function may have been changed and may contribute to increasing mortality after severe burn. However, at present, the underlying mechanisms of liver damage were no definite conclusions. The possible mechanisms were described as following. First, insufficient hepatic perfusion could contribute to liver damage.^[19]^ After burns, decreased hepatic blood flow was manifested by decreased portal venous blood flow, which was caused by the portal vein being more sensitive to catecholamine and significantly increased portal hypertension.^[15]^ Portal hypertension initiated by endotoxin has also been shown to induce hepatic microcirculatory disturbance.^[20]^ These pathophysiologic changes could cause liver damage. Second, burns may produce some chemical substances, including hepatotoxic substances. Hepatotoxic substance could cause hepatic metabolic change, increased inflammatory reaction and immune dysfunction.^[21,22]^ These factors play vital roles in combating and recovering from burned injury through multiple modulating pleiotropic pathways during the acute phase.^[15,23]^ Third, intrahepatic cholestasis has been thought to be one of important pathophysiologic processes after severe burn,^[24]^ which is the possible mechanism of liver damage. Finally, it has been suggested that liver damage is associated with increased hepatocyte cell death.^[21,23]^ However, the mechanism of programmed cell death in hepatocytes after burn has not been defined. ischemia-reperfusion may also contribute to hepatic apoptosis,^[24–26]^ It is presumed that the reduction in hepatic blood flow could lead to hepatic programmed cell death.^[27,28]^ In addition, proinflammatory cytokines such as interleukin-1(IL-1) and tumor necrosis factor (TNF)-α have been induced to promote apoptosis.^[29]^ Therefore, a decrease in splanchnic blood flow and an increase in proinflammatory cytokines initiate intracellular signaling pathways that were involved in increased hepatocyte apoptosis. Apparently, the liver is essential for post-burn outcome. Therefore, the attenuation of liver damage and restoration of liver function would improve the morbidity and mortality of severely burned patients.

It was found that the duration of peaked liver damage was specific for the second week and the time of peaked value for mortality and TBIL in non-survivors was also observed at the same time point. The underlying pathophysiological mechanism remains not understood. However, it is known that that hepatomegaly is one of important changes in response to burn injury, which could induce liver damage following burn injury.^[26]^ A prospective study with a large sample size has reported that the size of the liver in response to burns significantly increased, and the peak value was at the second week post-burn injury.^[5]^ This suggests that the time point of peaked liver damage occurred at the second week throughout the acute hospitalization after burns, and severe liver dysfunction may contribute to increasing mortality or non-survivors. The present study has a number of limitations. The sample size of the study was relatively small, and was limited to subjects from one accident and single-center. This was a retrospective study, and thus its clinical significance of liver dysfunction as a predictor of mortality in severe burns should be interpreted cautiously. Prospective cohort studies with a larger sample size throughout acute hospitalization after burns are needed to confirm these findings, and to determine the changes in liver size, weight, and hepatic protein synthesis.

In conclusion, the mortality rate among severely burned patients remains high, especially in patients with liver dysfunction during first 2 weeks. The great changes of liver function also occurred in first 2 weeks. The present study suggested that liver dysfunction would contribute to burn-related death in young adult patients with third-degree burns of over 90% TBSA during first 2 weeks. So liver dysfunction may have an effect on clinical outcomes of post-burn. In addition, measures to protect liver function and prevent from deterioration could be beneficial in improvement survival rate, especially during the first 2 weeks.

## Author contributions

**Conceptualization:** Yan Gong, Qiang Guo.

**Data curation:** Yan Gong, Xianming Long, Hua Xu, Xinjing Yang.

**Formal analysis:** Yan Gong, Xianming Long, Hua Xu, Xinjing Yang.

**Funding acquisition:** Qiang Guo, Yan Gong.

**Project administration:** Yan Gong, Xianming Long.

**Resources:** Qiang Guo, Yan Gong.

**Writing – original draft:** Yan Gong, Xianming Long.

**Writing – review & editing:** Qiang Guo.
